# Primary cardiac synovial sarcoma originating from the atrial septum and associated pulmonary infarction: a case report

**DOI:** 10.1007/s00432-024-05852-w

**Published:** 2024-08-20

**Authors:** Nie Xu, Kang Xie, Dong Xin, Zhonglei liang, Yongjun Zeng

**Affiliations:** 1 Department of Oncology, Chengdu First People’s Hospital, Chengdu, China; 2 Department of Pathology, Chengdu First People’s Hospital, Chengdu, China; 3 Department of Cardiovascular Surgery, Chengdu First People’s Hospital, Chengdu, China

**Keywords:** Cardiac synovial sarcoma, Intracardiac mass, Atrial septum, Pulmonary infarction, Cardiac surgery

## Abstract

**Background:**

Primary cardiac synovial sarcoma is a rare condition with limited treatment options for advanced stages. Surgery and chemotherapy are currently the mainstay treatments; however, survival rates remain low.

**Case presentation:**

A 64-year-old woman presenting with symptoms of chest tightness and shortness of breath was found to have an obstructive right atrial mass, along with pulmonary infarction and metastasis. She was ultimately diagnosed with advanced primary cardiac synovial sarcoma. Following surgery, the patient’s symptoms improved, and she underwent chemotherapy and anti-angiogenic therapy, but unfortunately, her survival time was only 8 months.

**Conclusion:**

This case report aims to enhance clinicians' understanding of the diagnosis and treatment of primary cardiac synovial sarcoma. Enhancing both survival outcomes and quality of life in individuals with primary cardiac synovial sarcoma continues to present a significant challenge.

**Supplementary Information:**

The online version contains supplementary material available at 10.1007/s00432-024-05852-w.

## Introduction

Cardiac malignancies can be classified into two types: primary cardiac malignancies and metastatic cardiac malignancies, with metastatic tumors being more common. The prevalence of primary cardiac tumors is 30 times less than that of metastatic tumors (Nunnery et al. 2020).The predominant pathological type is primary sarcoma (88.5%), with subtypes including angiosarcoma (40.4%), spindle cell sarcoma (5.4%), giant cell sarcoma (4.1%), and synovial sarcoma (4.3%) (Sultan et al. 2020).Less than 100 cases of primary cardiac synovial sarcoma have been documented in the literature.

## Case presentation

**Fig. 1:**
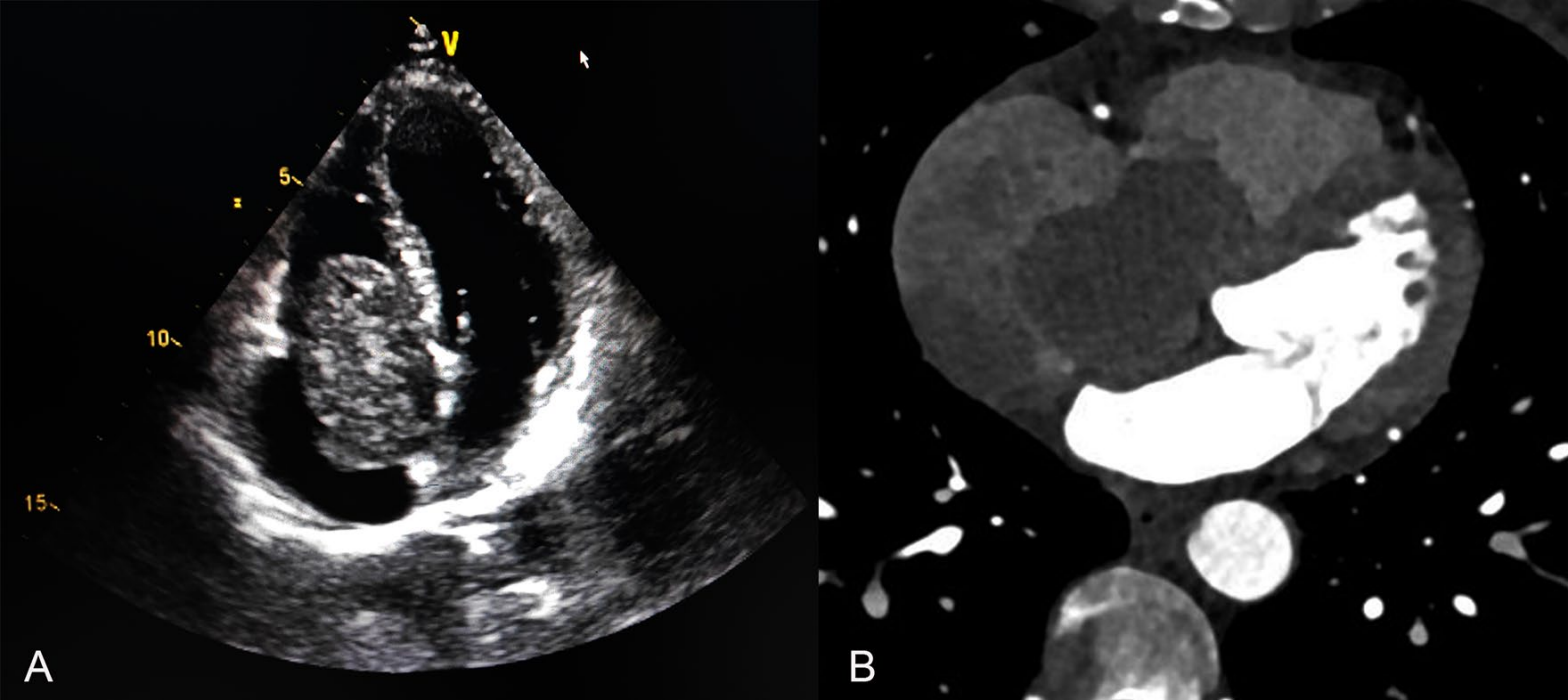
**A** Cardiac ultrasonography revealed an enormous tumor in the right atrium that is joined to the interatrial septum. **B** CT coronary angiography showed a mass extending from the right atrium through the interatrial septum to the right ventricle

A 60-year-old female patient exhibited symptoms of chest tightness, shortness of breath, palpitations, and fatigue, particularly worsening after physical activity. Cardiac ultrasound identified a mixed echo mass in the right atrium (Fig. 1A), along with right atrial enlargement, modest tricuspid regurgitation, and normal left ventricular systolic function. Coronary angiography demonstrated a massive filling defect in the right atrium, which extended from the right atrium through the interatrial septum to the right ventricle (Fig. 1B).Emboli were observed in the pulmonary artery within the middle and lower lobes of the right lung. No discernible abnormalities were detected in the coronary arteries. A chest CT scan revealed multiple nodules of varying sizes in both lungs (Fig. 4A). Additionally, blood tests, electrocardiogram, abdominal CT scan, lower limb vascular ultrasound, and brain MRI all showed no apparent abnormalities.

**Fig. 2:**
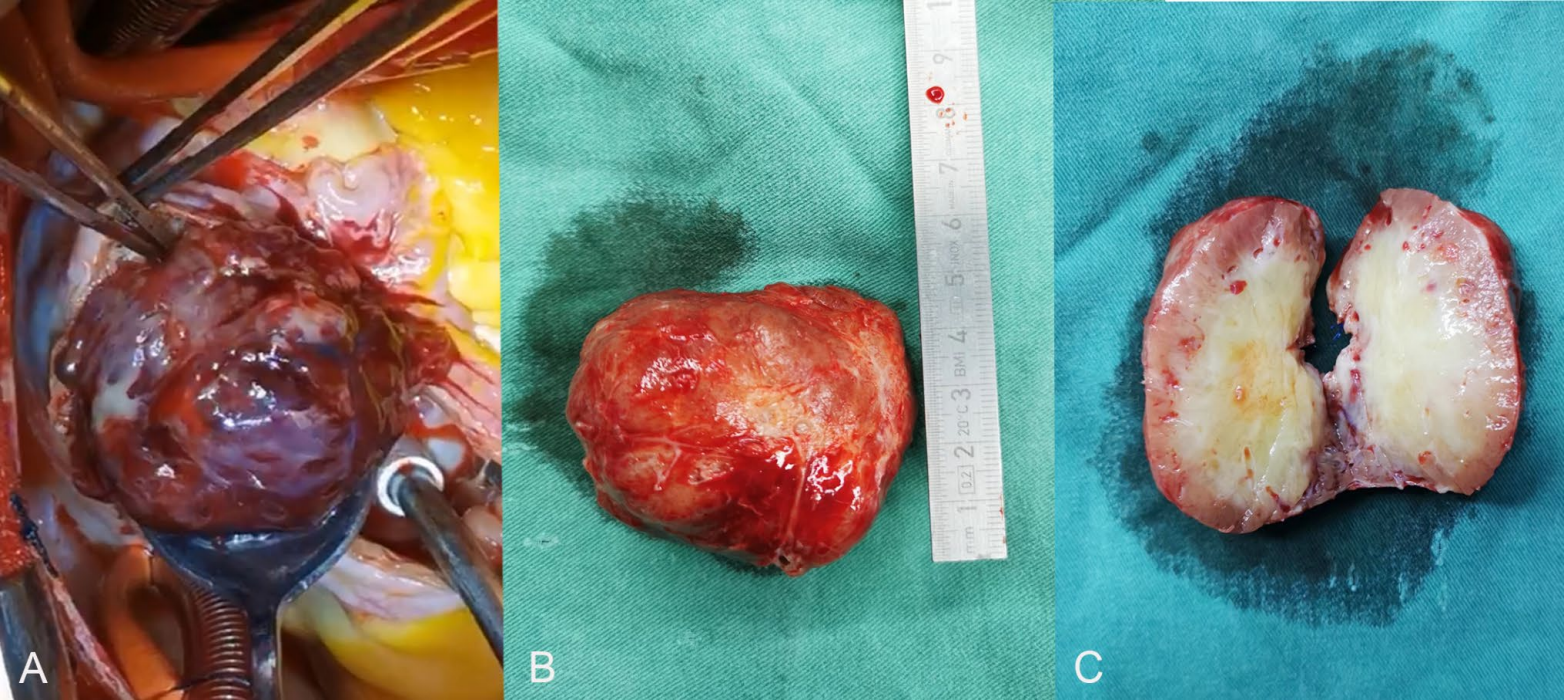
**A** Upon slicing the atrium, the tumor was seen to be loose and surrounded by both the right atrium and the right ventricle. **B **This is a huge hemorrhagic soft tissue mass (8.0 cm × 4.5 cm × 6.7 cm). **C** The sliced surface of the tumor is soft, with a gray-white center that appears necrotic and a reddish-brown margin

The skin and sternum were incised down the middle of the chest, followed by a longitudinal incision of the pericardium. After establishing extracorporeal circulation, it was observed that the size of the right atrial mass was approximately 8.0 cm × 4.5 cm × 6.7 cm (Fig. 2A). The tumor was loosely attached to the interatrial septum, and dilatation of the tricuspid valve annulus caused moderate to severe regurgitation. A cancer thrombus was identified in the right middle and lower pulmonary arteries. Following the complete resection of the right atrial tumor and a portion of the interatrial septal tissue (Fig. 2B, 2C), the main trunk of the right pulmonary artery was incised to remove the emboli from the middle and lower lobes of the right lung. Tricuspid annuloplasty was performed to reduce the size of the tricuspid annulus by cinching it with a continuous suture. The total operation time was 380 min. The postoperative condition was stable, and the patient was discharged from the hospital 10 days later.

**Fig. 3:**
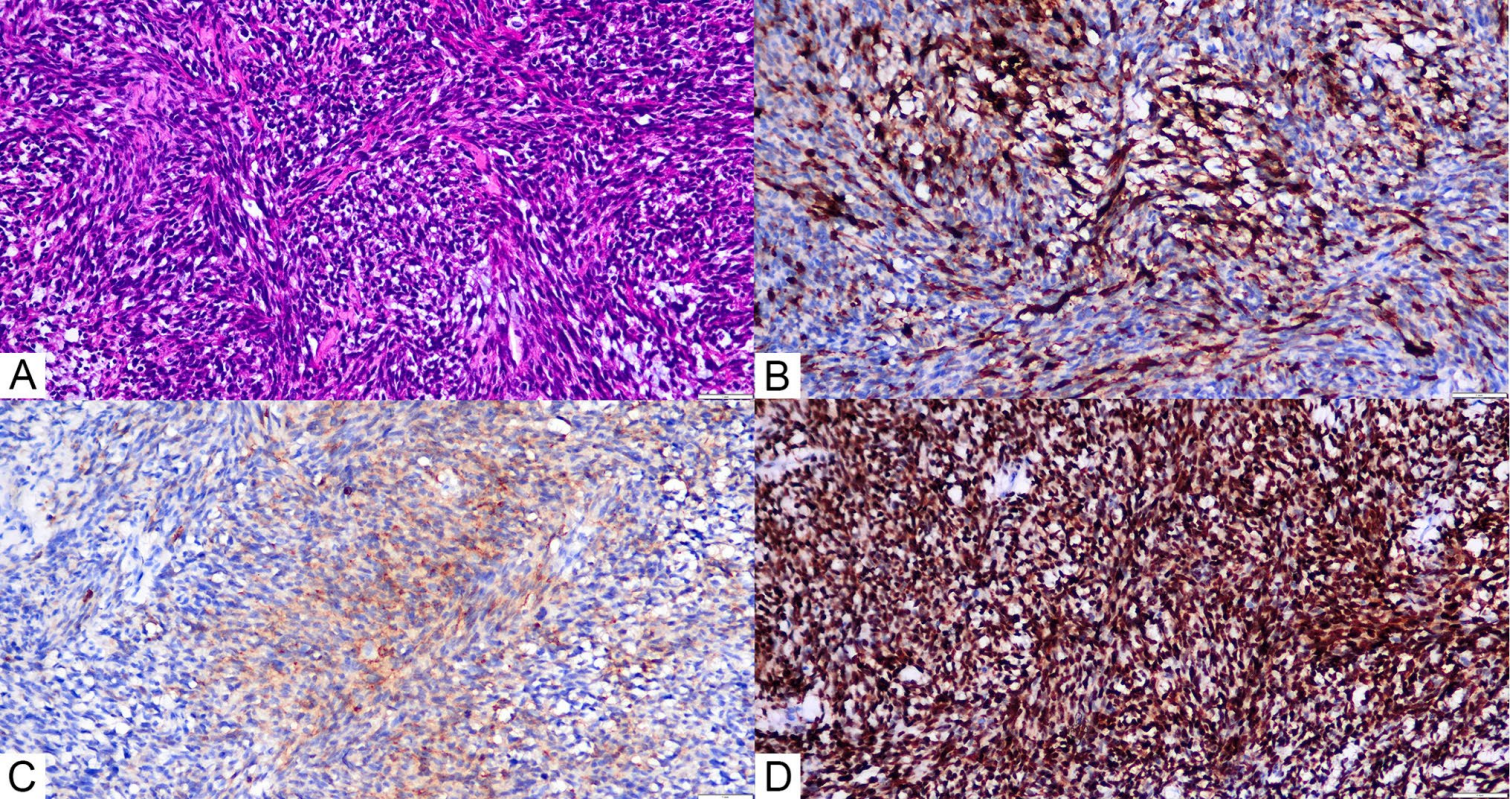
**A** (HE × 200)The histopathological investigation revealed a tumor composed of spindle-shaped monophasic cells grouped in interweaving bundles. **B** (IHC × 200) Calponin is expressed positive in the cytoplasm of most malignancies. **C** (IHC × 200) EMA is positive in the cytoplasm of focal malignancies. **D** (IHC × 200) TLE exhibited diffuse strong positive expression in the nucleus

**Fig. 4:**
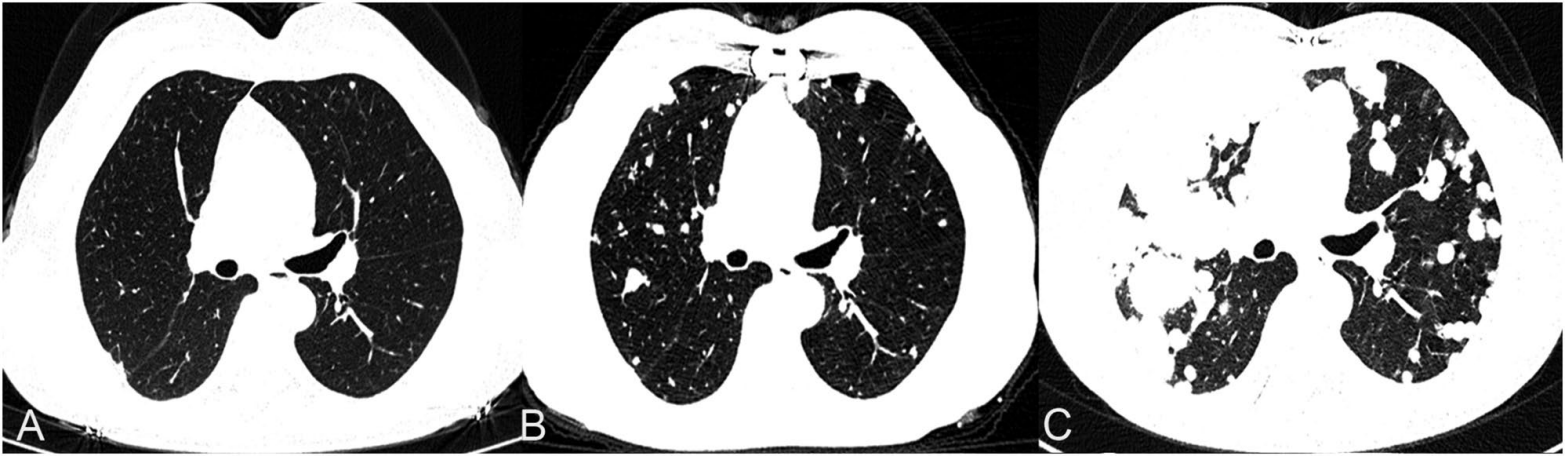
**A** A preoperative chest CT scan detected minuscule masses in the lungs. **B** Following 3 cycles of anlotinib treatment, subsequent chest CT scans showed an increase in subcentimeter nodules in both lungs compared to previous scans. **C** A chest CT scan after one cycle of second-line chemotherapy demonstrated a notable growth in size and number of metastatic lesions in both lungs, with patchy distributions, including a few lesions in the right hilus

Histological examination revealed single spindle cells grouped in bundles with alternating dense and loose regions, showing interstitial myxoid degeneration (Fig. 3A). Hemangiopericytoma-like structures were also found. The nuclei are uniform or oval in shape, mostly overlapping, darkly stained nuclei, indistinct nucleoli, and scant and poorly defined cytoplasm. An immunohistochemical investigation revealed positive tumor cells for EMA, Calponin, TLE-1, and P53 (Fig. 3B, C, D), with a Ki-67 proliferation index of 25%. Conversely, staining for S100 proteins, CD34, CD31, FLi-1, Myo-D1, smooth muscle antigens, and desmin was negative. Fluorescence in situ hybridization (FISH) detected the SS18:SSX translocation, validating the diagnosis of monophasic right atrial septal synovial sarcoma. Next-generation sequencing (NGS) was detected amplification of the KDR(kinase insert domain receptor), KIT(KIT proto-oncogene, receptor tyrosine kinase), and RICTOR(RPTOR independent companion of MTOR complex 2), as well as microsatellite stability(MSS). Following surgery, the patient received chemotherapy with a combination of ifosfamide (1400 mg/m2 d1–5) and anthracycline (25 mg/m2 d1-2, 21 days each cycle). After one cycle of chemotherapy, the neutrophil count declined to 0.05 × 109/L, while the platelet count dropped to 38 × 109/L. Granulocyte colony-stimulating factor was given, and the patient returned to normal following a platelet transfusion. Despite these interventions, the patient refused to continue chemotherapy due to significant bone marrow suppression (grade 4). Subsequent analysis through next-generation sequencing revealed amplification of the KDR gene, leading to the initiation of antiangiogenic therapy with anlotinib. Following 3 cycles of anlotinib treatment,a follow-up chest CT scan indicated more subcentimeter nodules in both lungs than earlier tests (Fig. 4B). Upon disease progression, the patient was transferred to single-agent liposomal doxorubicin (30 mg/m2 every three weeks) as second-line chemotherapy. Regrettably, the patient experienced hemoptysis one month later. To assess the lung condition, a chest CT scan was performed, which revealed a significant rise in the size and number of metastatic lesions in both lungs, which appeared in patchy distributions, including a few lesions in the right hilus (Fig. 4C). During hospitalization, the patient died unexpectedly of significant hemoptysis, with an 8-month survival time.

## Discussion

Synovial sarcoma is a mesenchymal tumor that accounts for 5%‐10% of soft tissue sarcomas (Kirkham et al. 2020; Nowicki et al. 2017). It primarily affects the limbs, with more rare occurrences in the heart.

The average age of patients was 32.5 years, with a higher prevalence in males (3.5:1 male to female ratio) (Hannachi Sassi et al. 2004). Patients commonly present with atypical clinical manifestations such as palpitations and dyspnea, which can often lead to misdiagnosis. Tumors can be detected using computed tomography (CT), magnetic resonance imaging (MRI), and cardiac ultrasound. The majority of cases (71%) are found on the right side of the heart, particularly in the right atrium (Wang and Li 2013), while only a small percentage (8.6%) occur in the left atrium (Coli et al. 2019).Our example came from the right interatrial septum, which is even more unusual. Cancer thrombosis on the right side of the heart increases the risk of pulmonary embolism. This patient’s tumor was big, spanning from the right interatrial septum to the right atrium and right ventricle, with the right atrium taking up the majority. A preoperative chest CT revealed many tiny nodules in both lungs. During the operation, it was discovered that the tumor had a loose texture, increasing the danger of tumor embolus dissociation during resection. As a consequence, the patient experienced pulmonary embolism and bilateral lung metastases.

Histologically, synovial sarcoma is a monomorphic spindle-cell sarcoma with variable epithelial differentiation. It can be categorized into three types: monophasic, biphasic, or poorly differentiated. The monophasic variety is made up entirely of spindle cells, whereas biphasic synovial sarcoma has both epithelial and spindle cell components (Jo and Fletcher 2014).Cardiac synovial sarcoma exhibits similar histological morphology. (Coli et al. 2019) retrospectively found that monophasic forms were approximately twice as common as biphasic forms in cardiac synovial sarcoma. Under the microscope, the patient was primarily made up of spindle cells, and the appearance was nearly identical. There were no zones of calcification or ossification, indicating a monophasic type. The cell nuclei were relatively uniform in shape, being spindle-shaped or oval and mostly overlapping, with darkly pigmented nuclei, indistinct nucleoli, and little or unclear cytoplasm.

There are no particular immunological markers for synovial sarcoma. The more sensitive markers are EMA, CK, CD99 (Bishop et al. 2015), Bcl-2, TLE-1 (Marchione et al. 2020), Calponin (Fisher et al. 2003), and Vimentin (Teng et al. 2021). A study reveals that H3K27Me3 and SOX10 are effective markers for neural soft tissue cancers (Sbaraglia and Dei Tos 2019). In this case, the absence of H3K27Me3, SOX10, and S100 can help exclude malignant peripheral nerve sheath tumors. Additionally, the lack of MyoD1 and S100 expression eliminates the possibility of rhabdomyosarcoma (Lak et al. 2021) and melanoma (Neubert et al. 2018), respectively, as these markers are known to be sensitive to those conditions. The patient has positive expression of TLE-1, Calponin, and EMA, supporting the diagnosis of synovial sarcoma.

According to molecular genetics, 95% of synovial sarcomas have a characteristic translocation of chromosome t (X: 18). The fusion of the SS18 (SYT) gene with one of the SSX genes (SSX1, SSX2, or SSX4) causes disease development. When synovial sarcoma is considered by immunohistochemistry and SS18 translocation is detected by FISH, synovial sarcoma can be diagnosed (Su et al. 2021).

The long-term survival percentage for primary cardiac malignant tumors is extremely low. According to the research of (Sultan et al. 2020), the 1-year and 5-year survival rates for primary cardiac malignant tumors were 45.3% and 11.5%, respectively, based on the US NCDB oncology database. Compared to the non-surgical group, the surgery group had considerably higher long-term survival rates (p < 0.0001).Cardiac synovial sarcoma is a highly malignant tumor with a lower incidence rate. There are currently no standardized treatment guidelines. Retrospective study (Coli et al. 2019) reported that the median survival time of 55 patients with cardiac synovial sarcoma was 27 months. Age, complete resection, and adjuvant chemoradiation were independent prognostic factors.The patient was found to have lung metastases. Due to the large size of the tumor and the risk of cardiac tamponade, palliative resection of the primary tumor is the only option to reduce the occurrence of severe complications.The most prevalent chemotherapy regimens are ifosfamide and doxorubicin; however, their efficacy is limited. In this example, the patient’s lung lesions grew slowly following chemotherapy. NGS testing reveals KDR amplification, which may result in overexpression of the VEGFR2 protein. We changed to anlotinib, which may have been sensitive to antivascular inhibitor medication, but the outcomes were unfavorable. As the disease advanced, pulmonary metastases infiltrated the major vessels near the right hilum of the lung, leading to massive hemoptysis and ultimately death. The patient had a survival time of 8 months with poor tolerance to chemotherapy, which was significantly lower than the median survival time reported by Coli et al. (27 months). It is important to note that the cases documented by Coli et al. predominantly did not involve distant metastasis and were successfully treated with complete resection, leading to a longer median survival time.

Here, We document a case of primary pericardial synovial sarcoma originating from the atrial septum and associated pulmonary embolism in a 60-year-old woman. This patient had pulmonary metastasis and pulmonary embolism at the time of diagnosis. He was in stage IV and theoretically lost the chance of surgery. However, due to the location of the primary tumor in the heart and the potential risks associated with blood flow obstruction, pulmonary embolism, and hemorrhage, a palliative tumor resection was performed. Given the intricate nature of cardiac synovial sarcoma and the lack of effective treatment options for patients in advanced stages, anti-angiogenic therapy continues to offer valuable insights into combating the disease. This emphasizes the necessity of exploring diverse treatment modalities to improve patient outcomes and potentially discover new avenues for therapeutic intervention. Research in this area remains crucial for advancing our understanding of the disease and developing more effective treatment strategies.

## Supplementary Information

Below is the link to the electronic supplementary material.

Supplementary file1 (DOCX 15731 KB)

Supplementary file2 (MP4 29798 KB)

## Data Availability

All data generated during this study are included in this published article.
