# Supplementary material for: Primary cardiac synovial sarcoma originating from the atrial septum and associated pulmonary infarction: a case report
Source: J Cancer Res Clin Oncol. 2024 Aug 20;150(8):392. doi: 10.1007/s00432-024-05852-w (PMC11335867; doi:10.1007/s00432-024-05852-w)
Supplement: Supplementary file 1 — Supplementary file1 (DOCX 15731 KB) [file 432_2024_5852_MOESM1_ESM.docx]

Supplementary Material

# 1.Supplementary Figures


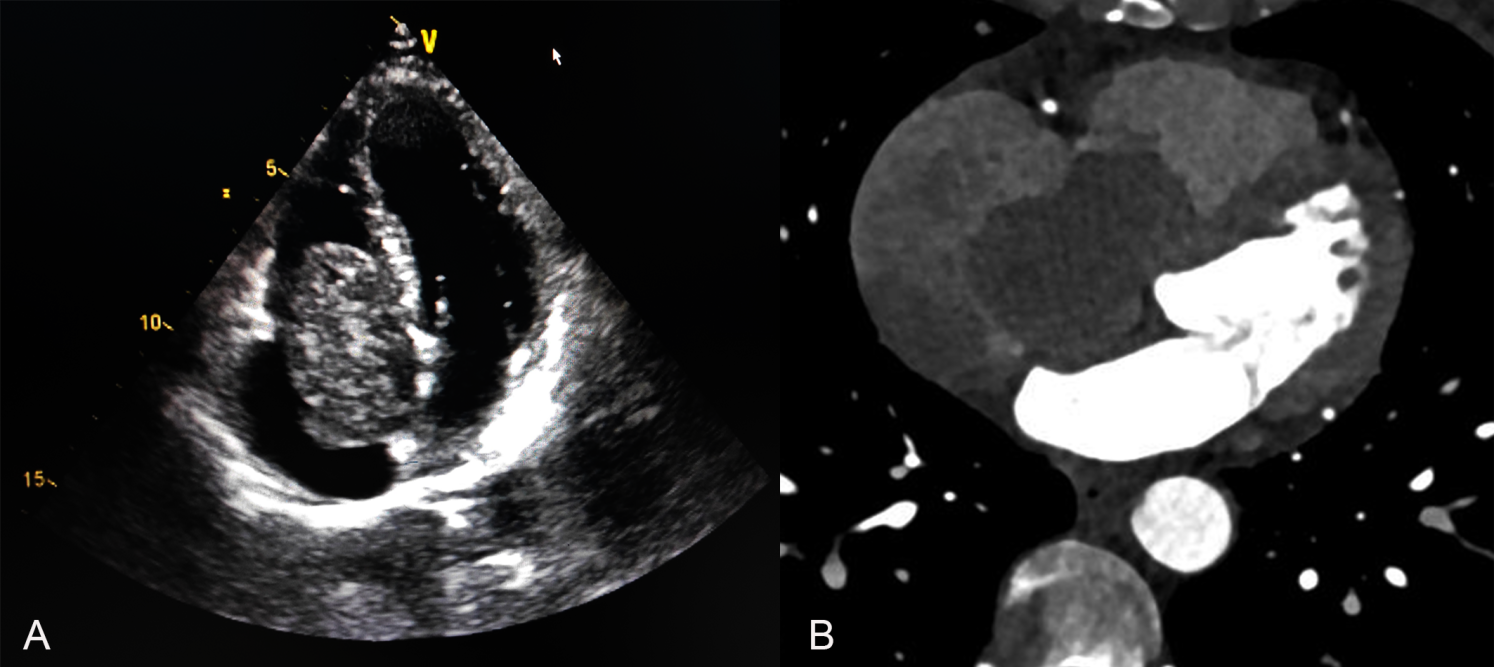


**Figure 1** (A) Cardiac ultrasonography revealed an enormous tumor in the right atrium that is joined to the interatrial septum.(B) Coronary angiography showed a mass extending from the right atrium through the interatrial septum to the right ventricle.


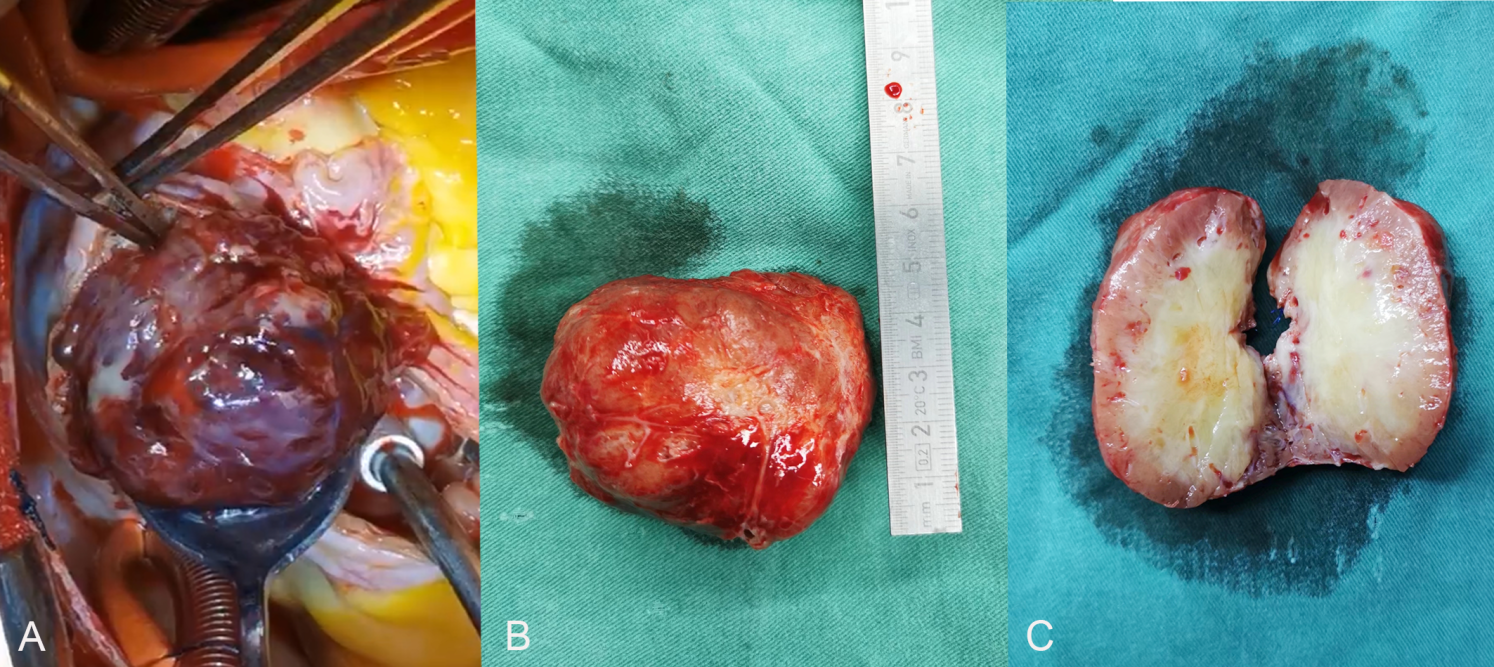


**Figure 2**  (A)Upon slicing the atrium, the tumor was seen to be loose and surrounded by both the right atrium and the right ventricle.(B)This is a huge hemorrhagic soft tissue mass(8.0cm×4.5cm× 6.7cm).(C) The sliced surface of the tumor is soft, with a gray-white center that appears necrotic and a reddish-brown margin.


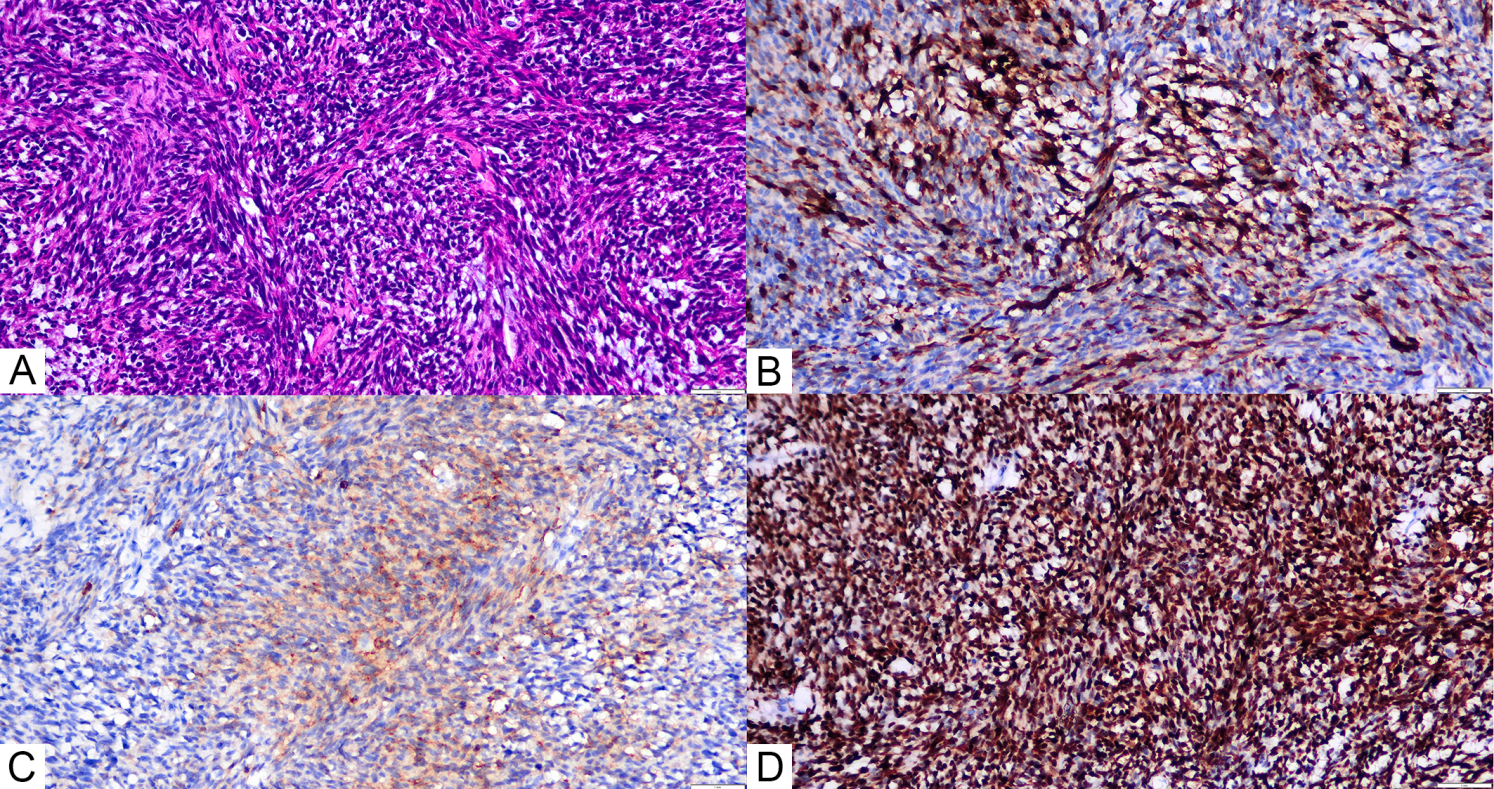


**Figure 3**  (A)(HE×200)The histopathological investigation revealed a tumor composed of spindle-shaped monophasic cells grouped in interweaving bundles.(B)（IHC×200）Calponin is expressed positive in the cytoplasm of most malignancies. (C) (IHC×200) EMA is positive in the cytoplasm of focal malignancies.(D) (IHC×200)TLE exhibited diffuse strong positive expression in the nucleus.


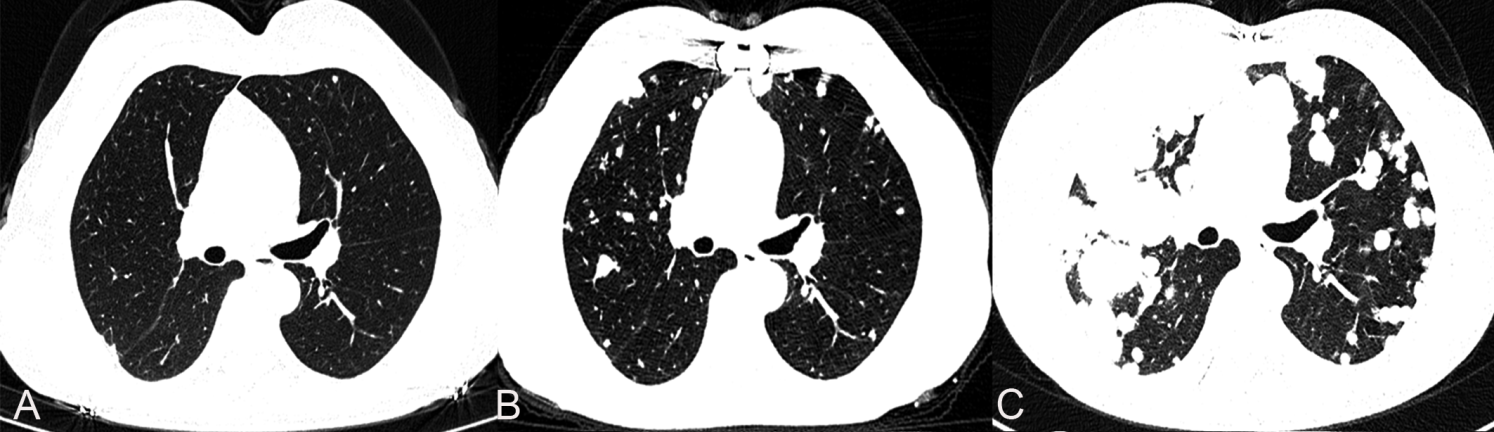


Figure 4 (A) A preoperative chest CT scan detected minuscule masses in the lungs. (B) Following 3 cycles of anlotinib treatment, subsequent chest CT scans showed an increase in subcentimeter nodules in both lungs compared to previous scans. (C) A chest CT scan after one cycle of second-line chemotherapy demonstrated a notable growth in size and number of metastatic lesions in both lungs, with patchy distributions, including a few lesions in the right hilus.
